# Supplementary material for: Caco-2 Cell Response Induced by Peptides Released after Digestion of Heat-Treated Egg White Proteins
Source: Foods. 2022 Nov 9;11(22):3566. doi: 10.3390/foods11223566 (PMC9689089; doi:10.3390/foods11223566)
Supplement: Supplementary file 1 [file foods-11-03566-s001.zip › foods-1990774-supplementary.pdf]

Supplementary Table S1. Primer pair sequences and conditions used for the analyses of gene expression.

Gene	Primer pairs	Reference	Cycling conditions
Gadph	*fw* 5' GAAGGTGAAGGTCGGAGTCAA 3'<br>*rv* 5' ACGTACTCAGCGCCAGCATC 3'	This work	*Pre-Incubation: 2 min 50 °C*<br>*Incubation: 10 min 95 °C*<br>**40 cycles**:<br>*Denaturation: 15 s 95 °C*<br>*Annealing/Extension: 45 s 62 °C*
Il33	*fw* 5′ GAGCTAAGGCCACTGAGGAA 3′<br>*rv* 5′ TGGGCCTTTGAAGTTCCATA 3′	[1]	
Il25	*fw* 5' CCAGGTGGTTGCATTCTTGG 3'<br>*rv* 5' TGGCTGTAGGTGTGGGTTCC 3'	[2]	
Tslp	*fw* 5' CTCTGGAGCATCAGGGAGAC 3'<br>*rv* 5' CAATTCCACCCCAGTTTCAC 3'	This work	
Il6	*fw* 5' TTCGGTCCAGTTGCCTTCTC 3'<br>*rv* 5' GAGGTGAGTGGCTGTCTGTG 3'	[3]	*Pre-Incubation: 2 min 50 °C*<br>*Incubation: 5 min 95 °C*<br>**40 cycles**:<br>*Denaturation: 15 s 95 °C*<br>*Annealing/Extension: 45 s 58 °C + 15 s 60 °C*
Il8	*fw* 5' AAGAAACCACCGGAAGGAAC 3'<br>*rv* 5' ACTCCTTGGCAAAACTGCAC 3'	[4]	*Pre-Incubation: 2 min 50 °C*<br>*Incubation: 10 min 95 °C*<br>**40 cycles**:<br>*Denaturation: 15 s 95 °C*<br>*Annealing/Extension: 45 s 62 °C*

fw, forward; *rv*, reverse

References

1. Tordesillas, L.; Goswami, R.; Benedé, S.; Grishina, G.; Dunkin, D.; Jarvinen, M.J.; Maleki, S.J.; Sampson, H.; Berin, M.C. Skin exposure promotes a Th2-dependent sensitization to peanut allergens. *J. Clin. Investig.* **2014**, *124*, 4965-4975.
2. Wang, Y.H.; Angkasekwinai, P.; Lu, N.; Voo, K.S.; Arima, K.; Hanabuchi, S.; Hippe, A.; Corrigan, C.J.; Dong, C.; Homey, B.; Yao, Z.; Ying, S.; Huston, D.P.; Liu, Y.J. IL-25 augments type 2 immune responses by enhancing the expansion and functions of TSLP-DC-activated th2 memory cells. *J. Exp. Med.* **2007**, *204*, 1837-1847.
3. Bunn, R.C.; Cockrell, G.E.; Ou, Y.; Thrailkill, K.M.; Lumpkin Jr, C.K.; Fowlkes, J.L. Palmitate and insulin synergistically induce IL-6 expression in human monocytes. *Cardiovasc. Diabetol.* **2010**, *9*, 73.
4. Townsend, D.M.; Lin-He, Y.M.; Xiong, Y.; Bowers, R.R.; Hutchens, S.; Tew, K.D. Nitrosative stress–induced S-glutathionylation of protein disulfide isomerase leads to activation of the unfolded protein response. *Cancer Res.* **2009**, *19*, 7626-7634.

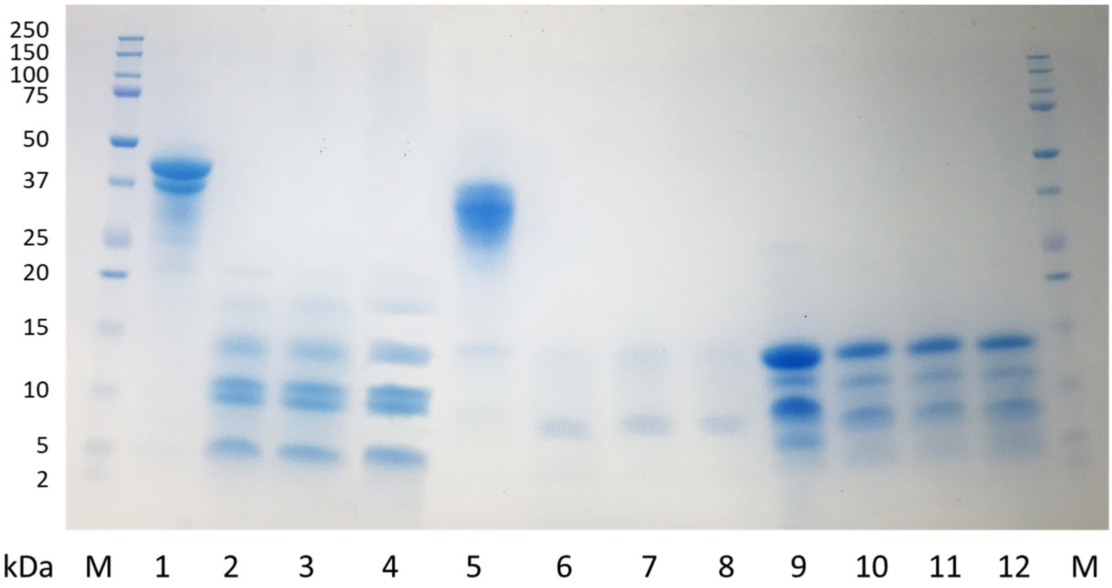

Supplementary Figure S1. SDS-PAGE patterns of OVA, OM, and LYS after in vitro gastroduodenal digestions. Electrophoresis was performed using Bis-Tris 12% acrylamide gel with reducing agents. Lanes: M, molecular mass marker (Bio Rad), ranging in molecular mass from 2 to 250 kDa; 1, OVA; 2, OVA digested; 3, OVA digested after treatment at 65 ˚C for 30 min; 4, OVA digested after treatment at 90 ˚C for 3 min; 5, OM; 6, OM digested; 7, OM digested after treatment at 65 ˚C for 30 min; 8, OM digested after treatment at 90 ˚C for 3 min; 9, LYS; 10, LYS digested; 11, LYS digested after treatment at 65 ˚C for 30 min; 12, LYS digested after treatment at 90 ˚C for 3 min.
